# Exploring the In Vitro Anti‐Inflammatory Effect of Citrus Fruit Hesperidin Supplementation

**DOI:** 10.1002/fsn3.70900

**Published:** 2025-09-07

**Authors:** Syed Yaseen Raza Zaidi, Aftab Ahmed, Gaurav Sanghvi, R. Roopashree, Aditya Kashyap, Nameer Khairullah Mohammed, Bushra Niaz, Farhan Saeed, Faiza Jamil, Muhammad Nadeem Akhter, Muhammad Afzaal, Catherine Tamale Ndagire

**Affiliations:** ^1^ Department of Nutritional Sciences Government College University Faisalabad Pakistan; ^2^ Marwadi University Research Center, Department of Microbiology, Faculty of Science Marwadi University Rajkot Gujarat India; ^3^ Department of Chemistry and Biochemistry, School of Sciences JAIN (Deemed to Be University) Bangalore Karnataka India; ^4^ Centre for Research Impact & Outcome, Chitkara University Institute of Engineering and Technology Chitkara University Rajpura Punjab India; ^5^ Department of Food Science, Faculty of Agriculture Tikrit University Tikrit Iraq; ^6^ Department of Food Science Government College University Faisalabad Pakistan; ^7^ University Institute of Diet and Nutritional Sciences University of Lahore Lahore Pakistan; ^8^ Department of Food Innovation and Nutrition Mountains of the Moon University Fort Portal Uganda

**Keywords:** anti‐inflammatory, antioxidant, citrus fruit, hesperidin

## Abstract

The aim of the present study was to assess the anti‐inflammatory effect of hesperidin. The research was conducted by optimizing the hesperidin extraction process from citrus peel powder, followed by characterization and nutrition profiling of citrus peel hesperidin extract. Citrus peel was collected from the local market and dried in a hot air oven. Then it was ground in a grinder to make powder. Then, the powder was extracted first with petroleum ether and then with methanol inside a Soxhlet apparatus. Then, the methanolic extract was filtered and subjected to evaporation in a rotary evaporator. Hesperidin was precipitated and purified with the help of acetic acid and dimethylformamide. The anti‐inflammatory and antioxidant perspective of hesperidin was assessed through NO_2_ production assay, assay of inflammatory cytokines, DPPH, and FRAP, respectively. For instance, in the ferric reducing antioxidant power assay, for 10 μM of hesperidin, the mean reduction value of three different tubes was observed as 3.36 ± 0.197. With the rise of the quantity of hesperidin, that is, 50, 75, and 100 μM, the mean values were observed as 5.48 ± 0.279, 7.5 ± 0.259, and 10.050 ± 0.832, respectively. Similarly, for the 2, 2‐diphenyl‐1‐picrylhydrazyl assay, the mean percentage reduction of the DPPH by hesperidin was 24 ± 0.5774, 35 ± 0.5774, 38 ± 0.57, and 40% ± 0.5% against the concentrations of 10, 25, 50, and 100 μM, respectively. As far as anti‐inflammatory activity is concerned, in the NO2 (Nitrite ion) production assay, the mean production of NO2 by LPS induction was 6.3366 ± 0.1 mM. However, co‐incubation of hesperidin with LPS in the concentrations of 10, 20, and 30 μM in the treatments has shown lesser productions of the NO2 as 4.8967 ± 0.5 μM, 3.6 ± 0.7 μM, and 2.8667 ± 0.5, respectively. Likewise, in the assay of IL‐8, IL‐1β and TNF‐α production assay, the mean production rate of cytokines was 0.6 ± 0.21 ng/mL, 0.47 ± 0.012 ng/mL, and 0.3633 ± 0.045 ng/mL according to the exposure rate of hesperidin as 0.01 mg/mL, 0.1 mg/mL, and 20 μM of the positive control drug PD98059. With a similar exposure rate of hesperidin and the positive control drug, the mean rate of production of IL‐1β was 0.0467 ± 0.079 ng/mL, 0.0367 ± 0.036 ng/mL, and 0.033 ± 0.021 ng/mL, respectively. The third cytokine TNF‐α had also shown similar patterns of inhibition against the same dose rate of hesperidin and PD98059. The mean value for the production of TNF‐α was 0.30 ± 0.18 ng/mL, 0.30 ± 0.091 mg/mL, and 0.1767 ± 0.084 ng/mL against the similar dose of hesperidin and PD98059. The inhibitory effect of hesperidin was more significant in IL‐8 as compared to IL‐1β and TNF‐α. Conclusively, hesperidin did prove to be a significant antioxidant and anti‐inflammatory agent.

## Graphical abstract

Citrus peel was collected from the local market and dried in a hot air oven. It was then ground in a grinder to make powder. Then, the powder was extracted first with petroleum ether and then with methanol inside a Soxhlet apparatus. The methanolic extract was filtered and subjected to evaporation in a rotary evaporator.

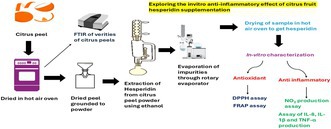

## 1 Introduction

The citrus plants, known as the Rutaceae family of plants, which includes 17 species, are distributed in tropical, subtropical, and temperate climates. These genera include oranges, kinnow, mandarins, lemons, and citrons grapefruit, which are significant fruits (Malik et al. 2021; Sorita et al. 2025). Characteristics of components of citrus fruits, which include oranges ( *Citrus sinensis* ), grapefruits (*Citrus paradisia*), tangerines ( *Citrus reticulata* ), limes ( *Citrus aurantifolia* ), and lemons ( *Citrus limon* ), are hesperidin and its derivatives. Citrus fruit varieties, parts of the fruit, the climate, and the stage of maturity all affect their composition (Liu et al. 2024; Pyrzynska 2022). During the processing of citrus, substantial amounts of solid waste are produced, which is primarily composed of peels and seeds. Citrus peel contains diverse types of organic compounds, which include polyphenols, vitamins, sugars, organic acids, fibers, and beneficial oils (Putnik et al. 2017; Sharma et al. 2017). In the sweet orange, hesperidin and naringin comprise more than 90% of the flavonoids (Erlund 2004). Because it does not accumulate, therefore, hesperidin is regarded as safe when consumed as a supplement or nutritional supplement, with nearly no side effects (Hajialyani et al. 2019).

Hesperidin was first extracted from citrus rinds by a French chemist, Lebreton (Roohbakhsh et al. 2015). The important sources of hesperidin are banana, bergamot, lemon, sweet lime, grapefruit, etc. (Huang et al. 2018). Overall, it can be found in the stem, leaves, petioles, flowers, and fruit sections of the Rubiceae genus and in the roots and entire grasses of cruciferous plants. Hesperetin is the aglycon form of hesperidin that is linked to the sugar molecule rutinose (6‐O‐α‐rhamnosyl‐D‐glucose). This is a disaccharide molecule present in the structure of hesperidin (Hajialyani et al. 2019). Hesperidin prevents damage to DNA and stress on the endoplasmic reticulum against alcoholic liver damage. Hesperidin also has numerous advantages such as anti‐inflammatory, antifungal, and antibacterial activities (Papoutsis et al. 2017; Shetty et al. 2016). Hesperidin is effective against liver damage. A study was conducted on rats that demonstrated that hesperidin had induced antioxidant defense systems in the rats (Çetin et al. 2016). Hesperidin has been very effective against hypertension in diabetic patients. Prolonged hypertension can cause various complexities such as stroke, heart attack, peripheral vascular, and cerebrovascular complications. Hesperidin consumption for 6 weeks can improve hypertension as compared to the placebo in type 2 diabetes (Homayouni et al. 2018).

Hesperidin has an important physiological role in the vascular endothelium and hence improves systolic and diastolic blood pressure. Vascular endothelium plays a significant role in balancing the vascular hemostasis by controlling the set balance of vasodilation and vasoconstriction (Rangel‐Huerta et al. 2015; Wunpathe and Kasorn 2025). Hesperidin treatment proved to be effective against isoproterenol‐induced ischemic rats (Selvaraj and Pugalendi 2012). Hesperidin has been proved effective against myocardial infarction in diabetic conditions. According to study results, hesperidin plays a significant role in reducing symptoms of myocardial infarction caused by STZ‐ISO (Agrawal et al. 2014). There are a number of methods to extract citrus flavonoids. Among them, organic solvent extraction, ultrasonic microwave, and supercritical fluid extractions are mostly used; every process has its own advantages and disadvantages. The most widely used method is organic solvent extraction because it is easy to use and efficient. Owing to the variations in the structures and polarity of flavonoids, different alcohols such as ethanol, methanol, acetone, ethyl acetone, and hot water are used (Kowalczyk 2024; Liu et al. 2021). Typically, dimethyl sulfoxide (DMSO), methanol, and ethanol, or their mixtures with water in varying ratios, are utilized for the extraction of hesperidin from citrus peel (Pyrzynska 2022). The current study is intended to optimize the hesperidin extraction process from citrus peels following the characterization and nutritional profiling of citrus peel hesperidin. In vitro anti‐inflammatory perspectives of citrus peel hesperidin were also studied.

## 2 Materials and Methods

### 2.1 Procurement of Raw Materials

Fresh orange peel was collected from the market of Government College University Faisalabad. Then, processing and analytical materials were obtained from the Department of Nutritional Sciences, Government College University Faisalabad. All the chemicals and instruments used were of standard grade. They were obtained from the laboratories of the Department of Nutritional Sciences, Government College University Faisalabad.

### 2.2 Preparation of Raw Material

The collected orange peel was dried in the hot air oven for a period of 2–3 days. Then, the dried orange peel was ground in the mechanical grinder.

### 2.3 Extraction of Hesperidin From Raw Materials

Using a Soxhlet Extractor, the orange peel powder was extracted through a variety of solvents, including acetone, methanol, and hexane. The Soxhlet extraction method was used using 100 g of orange peel powder and 750 mL of solvent (hexane, methanol, or acetone) at 50°C for 5 h. The extract was filtered after extraction. Whatman Filter Paper No. 2 was used to remove any peel particles from the extract. The filtered extract was then dried using a rotary evaporator at 60°C while being vacuum‐sealed. Until their next usage, the extracts were kept in a refrigerator at 4°C (Gotmare and Gade 2018). The process was optimized through control of temperature at 50°C, as a higher temperature could alter the nature of the compound of interest.

### 2.4 Purification of Crude Hesperidin

The crude hesperidin was then filtered and precipitated. Then, the dirty hesperidin was dissolved in 8 mL of dimethylformamide and then heated at temperature not more than 60°C–80°C. Then, 8 mL of distilled water was added drop by drop and left to cool at room temperature for 4 h. The precipitated crystals were pure hesperidin.

### 2.5 Characterization

The in vitro characterization of the hesperidin was carried out. Several antioxidant and anti‐inflammatory tests were carried out to assess the protective potential of hesperidin. Besides this, to assess the overall composition of citrus peel. Infrared spectroscopic analysis of three different varieties of citrus, that is, citrus sinenses, *citrus limetta* , and *citrus limon, was* done and explained.

### 2.6 Fourier Transform Infrared Spectroscopy (FTIR)

Fourier transform infrared characterization was done by adopting the method of (Dey and Sireswar 2021). FTIR spectrometer in the range of 600–4000 cm^−1^ and wavelength range of (700 nm–25 μm) was used.

### 2.7 Antioxidant Potential

To assess the antioxidant potential of the extracted citrus peel hesperidin, two major in vitro tests vis‐a‐vis antioxidant potential were conducted. Those include 2, 2‐diphenyl‐1‐picrylhydrazyl (DPPH) and ferric reducing antioxidant power (FRAP).

### 2.8 2, 2‐Diphenyl‐1‐Picrylhydrazyl (DPPH) Assay

With few modifications, the DPPH radical scavenging analysis was carried out using Sung‐Sook Choi et al. 2022 methodology. A 0.2 mL aliquot of the diluted sample was combined with 3.8 mL of the DPPH solution after 0.4 mM of DPPH was solubilized in 100% ethanol. After 30 min of reaction time, the absorbance at 525 nm was measured using a UV Spectrophotometer (S22, Biochrom, and Cambridge, UK) to determine whether the reactant color had stabilized. The amount of variation between samples and controls that the DPPH radical scavenging activity represented was expressed as a percentage.

### 2.9 Ferric Reducing Antioxidant Power (FRAP) Assay

A ferric reducing antioxidant assay was done to check the antioxidant potential of hesperidin. Fundamentally, in the FRAP assay, the antioxidant capacity is according to the capacity to reduce inside the redox linked colorimetric reaction. In the assay, the reducing capacity of the antioxidant substance is assessed in the ferric tripyridyltriazine complex, thereby reducing the blue color of the ferrous form. The activity of the antioxidant was detected at the absorbance of 593 nm. The compounds acting as antioxidants exert their reducing capacity by donating the hydrogen atoms to the ferric complex and consequently break the chain reactions of radicals. The higher absorbance reflects the higher antioxidant capacity of the compound, which ultimately was shown by the gradually increasing FRAP value in a dose‐dependent manner.

$$\begin{array}{c} \text{FRAP value}=\text{Absorbance sample}+\text{FRAP reagent}\\ \;-\text{Absorbance}\;\left(\text{FRAP reagent}\right) \end{array}$$

### 2.10 Anti‐Inflammatory Activity

To evaluate the in vitro anti‐inflammatory activity of citrus peel hesperidin, three different analyses were conducted. These include NO2 production, enzyme‐linked immunosorbent assay (ELISA) to assess the effect of hesperidin on the overproduced inflammatory cytokines, that is, IL‐8, IL‐1β and TNF‐α. The details of the procedure that were followed are given below.

### 2.11 NO2 Production

The presence of NO_2_ indicated an inflammatory burden on the body. It was measured by a specified reaction known as the modified Griess reaction. Cells were taken and incubated on 60 mm size tissue culture dishes. The media of the plates was changed with DMEM phenol red free and FBS after 24 h of incubation. After that, preincubation was done with various concentrations of hesperidin for a period of 30 min. Then lipopolysaccharide (LPS) of about 0.2 μg/mL was added to the media plates and then incubated at 37°C for 8 h. After that, Griess reagent was added to the media and then placed in a 96 well microplate. That microplate was then incubated for 15 min. For plotting the standard curve, NaNO_2_ solutions of 60 μM were introduced to the microplates as well. Then, by using the microplate reader, the plate was read at a wavelength of 540 nm. Then, concentrations were calculated by using the standard curves.

### 2.12 Assay of IL‐8, IL‐1β, and TNF‐α Production

To assess the in vitro anti‐inflammatory activity of citrus peel hesperidin, the efficacy of hesperidin assessed against the production of inflammatory cytokines was assessed through the enzyme‐linked immunosorbent assay (ELISA) test. The protocols and procedures followed were according to the method described by (Choi et al. 2007). Pretreatment of RAW 264.7 cells was done with the specified concentrations of hesperidin for a period of 30 min. Then, they were treated with PMA (50 μM) + A23187 (1 μM). Cell supernatants were collected, and concentrations of inflammatory cytokines were measured using ELISA. The ELISA plate ELISA was prepared by coating 96 well plates with mouse monoclonal antibody with specificity for the cytokines, that is, IL‐8, IL‐1β, and TNF‐α. Coated plates were washed with 0.05% PBS −20. Reagents that were used were incubated at 37°C for 2 h. In the subsequent steps, assay plates were exposed to the biotinylated mouse cytokines and ABTS substrate solution, which contained 30% H2O2. All the plates were read at 405 nm.

### 2.13 Statistical Analysis

The results were expressed as mean + SD of three replicates. Statistical analysis was performed using SPSS software, and one‐way ANOVA was used to analyze the results. The significance level of less than 0.05 was accepted.

## 3 Results and Discussion

Hesperidin was used as a central component of research. Hesperidin was extracted from the peels of citrus fruit. The samples of hesperidin were collected. Fourier transform infrared spectroscopy was done.

### 3.1 Fourier Transform Infrared Spectroscopy (FTIR)

**GRAPH 1:**
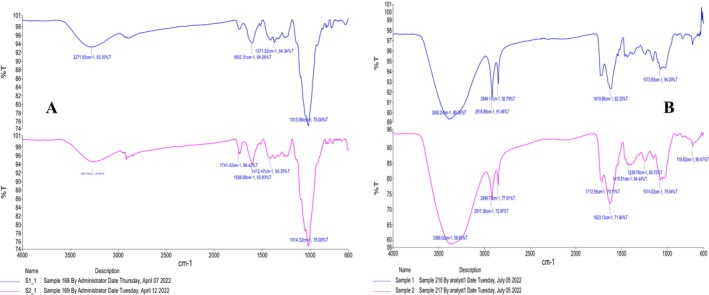
FTIR of *Citrus sinensis* peel powder and *Citrus limetta* peel powder. S1 presents *Citrus sinensis* and S2 presents *Citrus limetta* (A) and the FTIR of the *Citrus limon* peel. S1 presents sample no. 1, while S2 presents second sample (B).

The presence of the major elemental groups in the *citrus sinensis* peel (presented by sample 1) was analyzed by Fourier transform infrared spectroscopy. Patterns of peaks in Graph 1 (A) present various types of chemical groups that are present in sample 1. The first weak broad peak is at 3271.05 cm^−1^ which indicates the presence of O‐H acids. The second peak lies at 1632.31 cm^−1^, which indicates the presence of alkanes with C‐H groups. The third peak is a sharp weak peak that lies at 1371.52 cm^−1^ that indicates the presence of a carbonyl group. The fourth peak is a broad strong peak that lies at 1015.88 cm^−1^ which indicates the presence of a glycosidic ring C‐O‐C.

The Fourier transform infrared spectroscopy of *Citrus* *limetta* is presented as sample 2 in Graph 1 (A). The red line presents the peaks of the sample *Citrus* *limetta* peel powder. The first peak lies at 3257.70 cm^−1^ which indicates the presence of the O‐H acid group. The second peak is at 1741.42 cm−1. The third peak lies at 1598.99 cm−1. The fourth peak is a weak sharp peak that lies at 1412.47 cm−1. The fifth peak lies at 1014.32 cm−1, which indicates the presence of the glycosidic ring C‐O‐C.

Fourier transform infrared spectroscopy of *citrus limon* peel is presented in Graph 1 (B). The first peak is a broad strong peak that lies at 3382.29 cm^−1^ that indicates the presence of O‐H acid. The second and third peaks are sharp medium peaks that lie at 2916.89 and 2849.17 cm^−1^, respectively, which indicates the presence of C‐H alkanes. The fourth peak is a broad medium peak that lies at 1619.88 cm^−1^ that indicates cyclic alkanes. The fifth peak lies at 1072.83 cm^−1^. The second line of the graph presents the second sample of the *citrus limon*. The peaks are somewhat different from the peaks of the first sample. The first peak lies at 3366.02 cm^−1^. The second peak lies at 2917.35 cm−1. The third peak is at 2849.73 cm^−1^. Fifth and sixth peaks are at 1712.56 and 1623.12 cm^−1^. Then come sharp and weak peaks at 1415.51, 1239.78, 1074, and 718.82 cm^−1^. The nutritional values are assessed to determine the concentration of hesperidin and allied compounds present in various varieties of citrus peel. Moreover, it allows valuable insight to the readers concerning significant compounds present in the samples evaluated.

### 3.2 Ferric Reducing Antioxidant Power Assay

**GRAPH 2:**
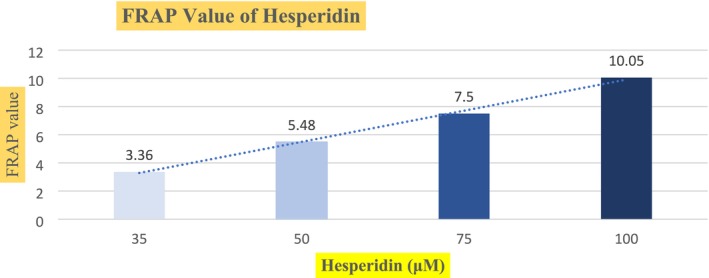
Effect of various concentrations of hesperidin on the FRAP reagent (insert here).

Graph 2 shows the mean value of ferric reducing antioxidant power of various concentrations of hesperidin. This is also known as an assay of reductive potential. It actually estimates the reductive potential of the antioxidant. The power of hesperidin is to reduce the iron that is present in the ferric reagent. Therefore, at the end of the experiment, the greater absorbance indicates the greater antioxidant power. The more reduction is indicated by the more bluish color of the reagent. Nevertheless, a light bluish color represents lesser activity of the antioxidant. The reducing power of hesperidin increased in a dose‐dependent manner. However, after a certain point, that is, at 100 μM, above that, the reducing power of the hesperidin did not increase. This is because the value of the reagent was controlled, and we were only increasing the hesperidin concentration. The more and more antioxidants added to the reagent, the maximum reduction of the reagent had been achieved, and therefore, an equilibrium had been achieved. The maximum absorbance was observed at the dose rate of 100. Above that, an equilibrium was established. Consequently, further addition of hesperidin did not reduce the ferric reagent.

At the initial concentration, 10 μM of hesperidin, the mean reduction value of three different tubes was observed as 3.36 ± 0.197. With the rise of the quantity of the hesperidin, that is, 50, 75, and 100 μM, the mean values were observed as 5.48 ± 0.279, 7.5 ± 0.259, and 10.050 ± 0.832, respectively. The difference in all the means reflects that our results were highly significant in all the tests. For every concentration, three tests were conducted, and their values were recorded. This increase in the reduction of the FRAP reagent shows that hesperidin is a powerful antioxidant in a dose‐dependent manner.

Our results are in agreement with (Guazelli et al. 2021), in which the ferric reducing antioxidant power of hesperidin has been reported in a dose‐dependent manner. Accordingly, (Pradeep et al. 2012) reported the antioxidant potential of hesperidin in FRAP of the gamma‐irradiated cells of mice. Hesperidin reduced oxidative stress in all types of body cells that were induced with oxidative stress by exposure to radiation. In a dose‐dependent manner, hesperidin reduced oxidative stress. Hence, the protective effect of hesperidin depends upon its exposure. Another study (Gorinstein et al. 2006) reported the reducing potential of hesperidin in the FRAP assay.

The results in our study are significant at 10.050 ± 0.11, as compared to the FRAP activity reported by (Gorinstein et al. 2006) as 1.69 ± 0.3 μmol Fe^2+^ μmol ^−1^. The high antioxidant capacity of the research might be due to the quality and varying types of oranges in different geographical areas. In a similar sense, Martinez et al. (2015) reported the antioxidant potential of hesperidin in FRAP. Although the hesperidin exposure resulted in a reduction of FRAP reagent in a dose‐dependent manner. However, significant results were observed at a much higher dose of 300 mg as compared to our dose of 30 μM. The mechanism behind such a vast difference is because of the presence of the HMC^−1^ cells in the research reported by (Martinez et al. 2015). Besides, the reaction kinematics of the active substance and the capacity to donate hydrogen ions highly depend upon the conditions in which the reaction proceeds. Oxidative stress damages all organs of the body (Adwas et al. 2019). Therefore, to reduce such damage, hesperidin shows its potential effect to reduce oxidative damage.

(Martinez et al. 2016) reported the antioxidant potential of hesperidin in the ultraviolet radiated cells in the FRAP assay. Their results are close to the values of our research. There was a slight difference of method as the antioxidant potential of hesperidin was assessed on the formulation of skin rather than the direct potential of hesperidin. However, the general reagent was the same that is, FRAP, and potential was assessed against it.

### 3.3 2, 2‐Diphenyl‐1‐Picrylhydrazyl Assay

2,2‐diphenyl‐1‐picrylhydrazyl is commonly known as DPPH. The reagent is known to be a highly stable free radical and hence an oxidant that can have the capacity to initiate chains of oxidizing reactions.

**GRAPH 3:**
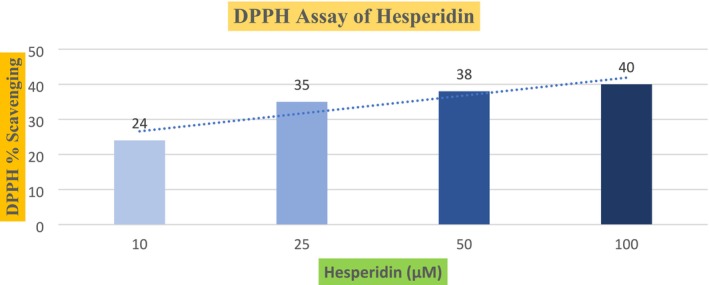
Antioxidant activity of various concentrations of hesperidin in the DPPH assay. (insert here)

Graph 3 shows the radical scavenging activity of citrus peel hesperidin in the 2,2‐diphenyl‐1‐picrylhydrazyl reagent. DPPH is a commercially available reagent. The presence of antioxidants reduces its dark purple color (Hajimahmoodi et al. 2014). 2,2‐Diphenyl‐1‐picrylhydrazyl is a stable free radical and is light sensitive. Therefore, all the experiments were done in the dark area. Four treatments or experiments with various concentrations of hesperidin reflected the high antioxidant capacity of hesperidin. Three independent experiments were done, and their scavenging activities were calculated as the means of the measurements. In all three experiments, the mean percentage reduction of the DPPH by hesperidin was 24 ± 0.5774, 35 ± 0.5774, 38 ± 0.57, and 40% ± 0.5% against the concentrations of 10, 25, 50, and 100 μM, respectively. The reduction of DPPH reagent increased with every rise in quantity with increasing concentration of hesperidin. Our results are similar to those presented by Binkowska (Binkowska, 2020), which reported the DPPH activity of hesperidin in a dose‐dependent manner. For 1, 3, and 7 mg of hesperidin, the IC50 inhibition percentages of hesperidin were 50.8 ± 0.1, 60.3 ± 0.2, and 75.5 ± 0.2, respectively. Relative to this, our results also suggested an increase in the percentage of scavenging activity with a rise in the dose of hesperidin. The fading of the dark purple color indicates the reduction of DPPH. At 10 μM, the color of the reagent was dark blue. While at 50 μM, the color changed to pale yellow. At 100 μM, the reagent becomes colorless. By denoting the hydrogen ions to the unstable DPPH reagent, hesperidin stabilized the compound. Our results were significant as there was a major difference between the means of all treatments.

The greater the reduction of the DPPH reagent, the greater the antioxidant activity of the reacting antioxidant. A study reported by (Cao et al. 2018) explains the high antioxidant activity of hesperidin, as the structure of hesperidin has loosely bound hydrogen ions which consequently allows it to easily donate hydrogen ions to the reacting substance. Similarly, (Wilmsen et al. 2005) reported the antioxidant activity of hesperidin. Compared to their reported data, the results of this research were more significant. Accordingly, (de Oliveira et al. 2013) reported the high scavenging activity of hesperidin in the DPPH assay of the serum cells of the rats suffering from oxidative stress. The aberrations in the in vitro and in vivo results might be due to the limited bioavailability of hesperidin in the subjects. Relative to this, our results also suggested an increase in the percentage of scavenging activity with a rise in the dose of hesperidin.

Briefly, the results and all the above‐mentioned studies suggest that hesperidin is a powerful antioxidant. The oxidative stress substances are short‐lived. Therefore, the radical scavenging power of a substance greatly depends upon the kinetics of the reacting substances. Jabbari and Jabbari (Jabbari and Jabbari 2016) reported the kinetics of hesperidin in relation to its reaction with DPPH solution in the presence of micelles of the cationic system of CTAB and the anionic system of SDS. A direct relation was observed between the reaction rate and the increase of hesperidin. However, that might be due to the presence of micelle systems which increase the hydrophobic interactions and electrostatic forces of the reacting atoms of the molecules of hesperidin and DPPH.

### 3.4 NO_2_ *(Nitrite Ion)* Production Assay

Inflammation is associated with all sorts of cancers, and nitric oxide radical (NO) has ostensibly been known to be high in nearly all types of cancers. Its signaling reduces the prognosis of the malignancies (Drehmer et al. 2022). In response to inflammatory stimuli, high nitric oxide (NO) is produced and hence mediates the inflammation and its destructive effects (Korhonen et al. 2005). Therefore, an estimation of NO production indicates the presence of inflammation. However, during severe stress conditions, nitric oxide undergoes oxidation and produces nitrite ions (NO2). Resultantly, high nitrite ions indicate less NO production and high inflammation.

**GRAPH 4:**
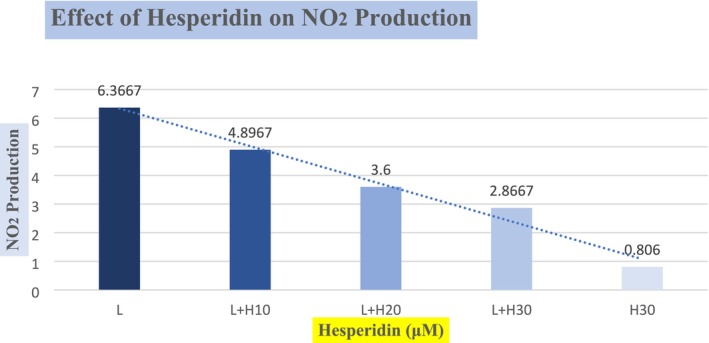
Potential of hesperidin to halt the production of nitrite ions. (Insert Here)

Graph 4 represents the production of NO_2_ in the LPS induced RAW 264.7 cells and the effect of various concentrations of hesperidin on the production of NO_2_. In the first sample column, the mean production of NO_2_ by LPS induction was 6.3366 ± 0.1 mM. However, co‐incubation with hesperidin with LPS in the concentrations of 10, 20, and 30 μM in the treatments has shown lesser productions of the NO_2_ as 4.8967 ± 0.5, 3.6 ± 0.7, and 2.8667 ± 0.5 μM, respectively. The difference in means shows that our results were significant. The results clearly reflect that hesperidin reduces the production of nitrite ions in a dose‐dependent manner.

Our results are in agreement with (Sakata et al. 2003), who reported the inhibitory effect of hesperidin against NO2 production. Similarly, (Kaur et al. 2006) reported the anti‐inflammatory activity by assessing the potential of hesperidin on the inhibition of NO2 production. According to (Kaur et al. 2006), hesperidin reduced the production of NO2 in a dose‐dependent manner. The mechanism behind the inhibition of NO2 is due to the ability of hesperidin to scavenge the free reactive oxygen that reacts with NO to produce NO2. (Saha et al. 2004), thereby leaving NO to mitigate the pro‐inflammatory cytokines. Accordingly (Korhonen et al. 2005), it reported a similar mechanism to explain the key role of hesperidin in inflammation. Nitric oxide, when present in large amounts, helps to reduce inflammation in almost all kinds of disorders.

### 3.5 Assay of IL‐8, IL‐1β, and TNF‐α Production Assay

Cytokines are numerous small proteins that play a significant role in controlling immune cell behavior. They are mostly produced by macrophages. They are the prominent inflammatory mediators. Among them, IL‐8, IL‐1β, and TNF‐α were our focus of research. All three of them are proposed to be involved in pathogenesis (Carballo‐Villalobos et al. 2017). Hence, their enhanced production indicates inflammation and causes depressive‐like behavior. Lipopolysaccharides cause inflammation and increase the levels of inflammatory cytokines (Li et al. 2016).

**GRAPH 5:**
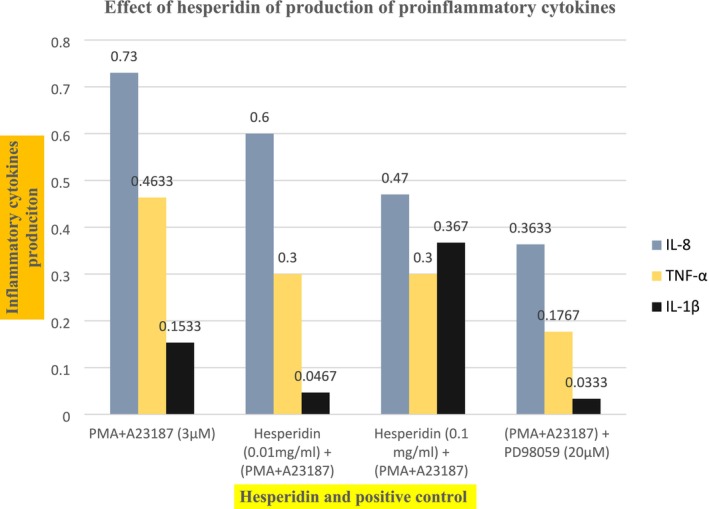
Effect of hesperidin on the production of proinflammatory cytokines IL‐8, IL‐1*β*, and TNF‐α. (Insert here)

Graph 5 shows data pertaining to the anti‐inflammatory activity of hesperidin against pro‐inflammatory cytokines IL‐8, IL‐1β, and TNF‐α, respectively. The data from the tests show that the presence of PMA + A23187 induced inflammation by increasing the production of the cytokines IL‐8, IL‐1β, and TNF‐α. However, co‐treatment with hesperidin at various concentrations led to a significant decrease in the production of these cytokines in a dose‐dependent manner. Although the efficacy of hesperidin was visible in all three cytokines Nevertheless, the most significant inhibitory rate was observed in IL‐8. The mean production rate of cytokines was 0.6 ± 0.21 ng/mL, 0.47 ± 0.012 ng/mL, and 0.3633 ± 0.045 ng/mL according to the exposure rate of hesperidin as 0.01 mg/mL, 0.1 mg/mL, and 20 μM of the positive control drug PD98059. With a similar exposure rate of hesperidin and the positive control drug, the mean rate of production of IL‐1β was 0.0467 ± 0.079 ng/mL, 0.0367 ± 0.036 ng/mL, and 0.033 ± 0.021 ng/mL, respectively. The third cytokine TNF‐α had also showed similar patterns of inhibition against the same dose rate of hesperidin and PD98059. The mean value for the production of TNF‐α was 0.30 ± 0.18 ng/mL, 0.30 ± 0.091 mg/mL, and 0.1767 ± 0.084 ng/mL against the similar doses of hesperidin and PD98059. The inhibitory effect of hesperidin was more significant in IL‐8 as compared to IL‐1β and TNF‐α. The difference of means shows that the results were highly significant.

Accordingly (Li et al. 2016), it was reported that the effect of hesperidin is in the inhibition of IL‐1β, TNF‐α, and IL‐6 in the LPS‐induced inflammatory cells of mice. Their results are in accordance with our results, as the inhibitory rate of hesperidin was increasing in a dose‐dependent manner. Our results are in agreement with the results reported by (Carballo‐Villalobos et al. 2017), who reported that treatment with hesperidin reduces the inflammatory cytokines that are released in neurotoxicity. Accordingly (Yeh et al. 2007), it was reported that the efficacy of hesperidin is in relation to the inhibition of pro‐inflammatory cytokines TNF‐α and IL‐1β in the LPS‐induced inflammation of alveolar cells. Hesperidin doses up to 100 μM were proved to be the most significant. Similarly, (Moon and Kim 2012) reported the capacity of hesperidin to inhibit the concentration of pro‐inflammatory cytokines in HaCaT cells. The LPS‐induced inflammation was mitigated in a dose‐dependent manner. Within the same perspective, (Gur et al. 2022) and (Bussmann et al. 2022) have reported the role of hesperidin as a mediator of pro‐inflammatory cytokines, thereby justifying the anti‐inflammatory role of hesperidin. The mechanism behind this capacity of hesperidin is its ability to halt the NF‐KB pathway. Suppression of NF‐KB results in decreased production of pro‐inflammatory cytokines.

## 4 Recommendations and Conclusion

Based on the above research, it can be concluded that hesperidin is a potent antioxidant and anti‐inflammatory compound. Its broad‐spectrum activity and high nutritional value suggest that it can be used as a nutraceutical. In the present study, hesperidin was extracted from citrus peel because citrus peel has the highest percentage of hesperidin as compared to other components of citrus. Pakistan is one of the largest producers of citrus; therefore, raw materials for producing hesperidin are available in ample amounts. Although a plethora of research has already been done on hesperidin. However, further research is needed to increase its availability and explore more nutritional benefits. There are certain limitations in terms of the scope of the research. The research solely explores the in vitro activity of the hesperidin and is unable to provide valuable insights into the possible effects of hesperidin in in vivo trials. While the conducted assays show significant results, they are unable to unveil the underlying molecular mechanisms.

It is recommended that subsequent research should include in vivo trials on humans in general as well as in specific conditions. It is also recommended that new and more efficient methods should be explored to maximize hesperidin production. High nutritional value pertaining to hesperidin suggests that it can also be used as a supplement for general health benefits. Further research in this domain may unveil other hidden benefits of hesperidin.

## Author Contributions

**Syed Yaseen Raza Zaidi:** software (equal). **Aftab Ahmed:** validation (equal). **R. Roopashree:** formal analysis (equal). **Aditya Kashyap:** formal analysis (equal). **Faiza Jamil:** conceptualization (equal).

## Conflicts of Interest

The authors declare no conflicts of interest.

## Data Availability

Although the authors have provided the required data, additional data can be provided on demand.
